# Knowledge, attitudes, and practices of oil and salt intake and related influencing factors in Southwestern China

**DOI:** 10.3389/fnut.2024.1334977

**Published:** 2024-11-06

**Authors:** Laixi Zhang, Qi Xu, Ke Jiang, Zhourong Li, Yaqi Wen, Zhichuan Hu, Changxiao Xie, Zumin Shi, Manoj Sharma, Yong Zhao

**Affiliations:** ^1^School of Public Health, Chongqing Medical University, Chongqing, China; ^2^Research Center for Medicine and Social Development, Chongqing Medical University, Chongqing, China; ^3^Research Center for Public Health Security, Chongqing Medical University, Chongqing, China; ^4^Nutrition Innovation Platform-Sichuan and Chongqing, School of Public Health, Chongqing Medical University, Chongqing, China; ^5^Department of Nutrition and Food Hygiene, West China School of Public Health, Sichuan University, Chengdu, Sichuan, China; ^6^Human Nutrition Department, College of Health Sciences, QU Health, Qatar University, Doha, Qatar; ^7^Department of Social and Behavioral Health, School of Public Health, University of Nevada, Las Vegas (UNLV), Las Vegas, NV, United States; ^8^Department of Internal Medicine, Kirk Kerkorian School of Medicine, University of Nevada, Las Vegas (UNLV), Las Vegas, NV, United States; ^9^Chongqing Key Laboratory of Child Nutrition and Heath, Children's Hospital of Chongqing Medical University, Chongqing, China

**Keywords:** intake of oil and salt, knowledge, attitude, practice, Southwestern China

## Abstract

**Objects:**

Excessive oil and salt consumption is a public health issue, notably in China where intakes surpass WHO guidelines. The present study aims to examine the knowledge, attitudes, and practices of Southwestern China residents regarding oil and salt and explore the influencing factors.

**Methods:**

This study used convenience sampling to collect data from 7,367 participants aged 18–75 in the Sichuan, Chongqing, Yunnan, and Guizhou regions of China via on-site face-to-face surveys. Descriptive statistics and generalized linear models were used to analyses knowledge, attitudes, and practices about oil and salt intake and their influencing factors among residents of Southwestern China.

**Results:**

In Southwestern China, residents of Guizhou Province exhibited poor KAP regarding oil and salt. There were urban–rural differences in Yunnan, Sichuan, and Chongqing, and residents living in towns and cities were the favored factors for KAP scores. Groups engaged in self-employment/sales and freelance were risk factors for KAP score. Individuals with higher education was a favorable factor for KAP score. In Yunnan, Sichuan, and Chongqing groups with preference of salty tastes were favorable factors in KAP score. Diabetic patients were more likely to score low on oil and salt-related KAP performance.

**Conclusion:**

In Southwestern China, residents of Guizhou Province displayed poor results in their KAP regarding oil and salt. The region of the province, ethnicity, urban and rural residence, education, taste preference, and prevalence of chronic diseases were the influencing factors of oil and salt-related KAP scores.

## Introduction

Edible salt (NaCl) and cooking oil play a key role in enhancing the taste and texture of food and are major components of food and important flavoring agents. However long-term adherence to a high-fat, high-salt diet has been identified as a risk factor for the onset and progression of chronic diseases (1).

A meta-analysis of research on salt intake and cardiovascular disease found that every 5 g increase in daily salt intake was associated with a 17% increase in the risk of total cardiovascular disease and a 23% increase in the risk of stroke (2). He et al. (3) discovered that reducing salt intake modestly led to a 20% reduction in the risk of cardiovascular disease and 73 fewer cases of stroke. Prolonged consumption of a high-fat diet not only contributes to the obesity epidemic but also to the progression of a variety of metabolic diseases (4, 5), including type 2 diabetes, atherosclerosis, hypertension, and stroke (6, 7). Long-term high-salt and high-oil intake will also change the composition of the intestinal microbiota, which in turn affects the phenotype and function of CD4 T-cells in the intestinal tract (8). This phenomenon leads to increased susceptibility to infections inside and outside of the intestinal tract, thereby increasing the risk of developing chronic autoimmune diseases (9).

The Dietary Guidelines for Chinese Residents (2022 Edition) suggest that the daily salt intake of adults should be no more than 5 g, and cooking oil intake should be 25–30 g (10). According to the Report on Nutrition and Chronic Disease Situation of Chinese Residents (2020), China’s dietary fat energy supply ratio has continued to increase, and the intake of edible oil and edible salt is higher than the recommended value (11). According to the survey data, the China’s *per capita* daily intake of cooking salt is as high as 11 g/d (12), and the *per capita* daily intake of cooking oil is 41.8 g/d (13), which is higher than the recommended intake of the World Health Organization (14). The primary way for intake of salt and cooking oil in China is distinct from that of prepared foods in other developed countries, with most of the dosage being added during the cooking process according to personal taste preferences (15, 16). The Southwest region (Sichuan, Chongqing, Guizhou, and Yunnan), under the influence of regional, climate, and ethnic characteristics, has maintained a diet with emphasis on taste for a long time, leading to the problem of excessive intake of oil and salt in this region (17). The prevalence of chronic diseases is higher in Southwestern China than in the central and coastal regions (18). Meanwhile, a study has shown that the age-standardized cancer and coronary heart disease rates increased most substantially in Southwestern China from 2007 to 2016 (19).

Knowledge, attitudes, and practices (KAP) are key elements in facilitating individual behavioral changes by improving cognitive understanding and shaping beliefs and attitudes that lead to behavioral change (20). Haron et al. (21) reported that although the vast majority of study participants had positive attitude toward healthy salt intake, their knowledge and practice of healthy salt intake were not at a satisfactory level. In a study of urinary sodium excretion among healthcare workers in Malaysia, individual KAP regarding salt intake and health-related issues influenced salt intake (22). A study in Shandong, China showed that people with unfavorable attitudes toward sodium reduction were less likely to reduce their sodium intake (23). In research on oil and salt intake in the Iranian population, only 32% were aware of the dangers of excessive consumption of animal oils, and urban households had significantly higher levels of knowledge about oil and salt than rural households (24). In another study on dietary fat intake among adolescents, only 47.7% of students, 48.2% of parents were aware that frying is not a healthy method of food preparation (25).

Previous works focused on the disease risks of excessive oil and salt intake and examined pathological mechanisms involved in the development of various diseases. However, research on public perceptions of oil and salt consumption and related factors is still relatively limited, especially in the southwestern region of China, which has a long history of excessive oil and salt diets. Differences in KAP have been studied to help in the construction and implementation of educational strategies and public policies that promote targeted behavior change (26). Collection, analysis, and evaluation of KAP related to oil and salt intake in the population are important to construct effective salt and oil reduction strategies. Therefore, the purpose of the study is to investigate the knowledge, attitudes, and practices of residents in Southwestern China who have long existed previous works focused on the disease risks of excessive oil and salt intake and examined pathological mechanisms involved in the development of various diseases. However, research on public perceptions of oil and salt consumption and related factors is still relatively limited, especially in the southwestern region of China, which has a long history of excessive oil and salt diets eating habits, and to explore the influencing factors.

## Methods

### Study design and sample

This study adopted a cross-sectional design and conducted from February to May 2021 in Yunnan, Guizhou, and Sichuan provinces and Chongqing Municipality in Southwestern China. We recruited investigators from six colleges and universities selected from four provinces in Southwestern China. After screening and uniform training, 252 investigators conducted face-to-face field surveys using paper-based questionnaires in households and communities in each district.

The inclusion criteria for this study were as follows: (a) age of 18–75 years, (b) local resident at least 3 years, and (c) ability to understand the contents of the questionnaire and fill in carefully. Uncooperative and cognitively impaired individuals were excluded. Based on the fact that the awareness rate of dietary nutrition among Chinese adult residents surveyed in 2015 was 21.1% and considering sampling errors and invalid questionnaires, the sample size was calculated to be 6,960. A total of 8,535 residents participated. After excluding outliers and missing values, 7,367 participants were included in the analysis. All participants were informed about the study and provided consent before filling out the scale anonymously. This study has been approved by the Ethics Committee of Chongqing Medical University (approval number: 2021041).

### Data collection

Data were obtained from the Questionnaire of dietary knowledge, attitudes, and practices of residents Southwestern China in the Dietary Practices and Dietary Culture Survey of Chinese Nutrition Society. The Cronbach’s *α* coefficient was calculated to be 0.825. In conducting the questionnaire survey, the participants were screened strictly according to the inclusion and exclusion criteria, and the investigators received unified training and passed the assessment.

The questionnaire consists of two parts: socio-demographic characteristics and basic knowledge of KAP for intake of oil and salt. Sociodemographic characteristics include (1) gender (male/female), (2) age, (3) height (self-reported), (4) weight (self-reported), (5) ethnicity (Han/minority), (6) residence (rural/urban), (7) region (Guizhou Province/Yunnan Province/Sichuan Province/Chongqing City), (8) long-term residents (yes/no), (9) occupation (animal husbandry and fishery/student/self-employed individual/sales/freelance), (10) education level (primary school and below/junior high school/senior high school/secondary technical school/junior college and above), (11) taste preference (light/sour/sweet/spicy/salty), (12) diagnosis of high blood pressure (yes/no), (13) diagnosis of diabetes (yes/no), and (14) diagnosis of gout (yes/no).

The KAP for intake of oil and salt by residents Southwestern China includes the following aspects: knowledge part: (1) guideline recommended daily intake of edible oil, (2) recommended daily salt intake, and (3) diet is the most direct and closely related to hypertension; attitudes part: (1) when cooking, I used vegetable oil instead of some animal oil (such as lard) and (2) condiments other than salt, I added extra salt intake; practices part: (1) how often you ate smoked products, (2) how often you eat cured products, (3) how often you eat hot pot each month, (4) in your daily diet, you consciously reduced the intake of cooking oil, and (5) made a conscious effort to reduce salt intake in your daily diet.

### Data processing

The age categories are delineated as follows: 18–25 years as one category, 26–45 years as another category, and >45 years as a third category. Body mass index (BMI) was calculated based on the self-reported height and weight of the survey respondents (weight/height^2^) and was classified as underweight (≤18.5 kg/m^2^), normal (18.5 kg/m^2^ ≤ BMI < 24 kg/m^2^), overweight (24 kg/m^2^ ≤ BMI < 28 kg/m^2^), and obesity (BMI ≥ 28 kg/m^2^) (27). Occupations were divided into five groups (animal husbandry and fishery, student, self-employed individual/sales, freelance, and employee). Education level was divided into low (primary school and below/junior high school), medium (senior high school/secondary technical school), and high (junior college and above). Taste preferences were divided into five categories (light/sour/sweet/spicy/salty).

Ten questions about the oil and salt intake were asked from residents Southwestern China: three questions about knowledge, two questions about attitudes, and five questions about practices. In this context, a correct response in the knowledge section was assigned a value of 1 point, while an incorrect response received 0. Attitudes and practices were assessed using a five-point Likert scale. Within the attitudes section, each question was rated as follows: “Strongly agree” received 5 points, “Agree” received 4 points, “Not sure” received 3 points, “Disagree” received 2 points, and “Strongly disagree” received 1 point. In the practices section, each question was scored as follows: “Never” at 1 point, “Occasionally” at 2 points, “Sometimes” at 3 points, “Often” at 4 points, and “Always” at 4 points; “Occasionally” was assigned 2 points, “Sometimes” 3 points, “Often” 4 points, and “Every day” 5 points; “≤ 1 time” was scored at 5 points, “2–3 times” at 4 points, “4–5 times” at 3 points, “6–7 times” at 2 points, and “≥8 times” at 1 point. The score was computed and standardized on a 100-point scale.

### Statistical analysis

Statistical analysis used frequencies and proportions (%) to describe categorical variables and mean ± standard deviation (SD) to describe continuous variables. The study employed a chi-square test for categorical variables and analysis of variance (ANOVA) for continuous variables to demonstrate variations in scores and responses for knowledge, attitudes, and practices across the four southwestern provinces and cities. T-test and ANOVA were utilized to evaluate differences in socio-demographic characteristics on KAP scores. Generalized linear models were used to assess the association between socio-demographic characteristics and KAP scores. Data were entered on EpiData software and analyzed with STATA version 17.0. Statistical significance was determined using a *p*-value of less than 0.05 (two-tailed).

## Results

### Basic demographic features

Of the 8,535 respondents who agreed to participate in the questionnaire survey, 7,367 study subjects were included after the implementation of the inclusion–exclusion criteria. The detailed basic demographic characteristics are tabulated in Table 1. The average age of the participants was 35.48 ± 14.41 years, with a high percentage of Han Chinese at 88.24% and the minority at 11.76%. The proportion of participants who lived in rural areas was 38.51%, and that in urban areas was 61.49%. The higher share of students in the occupational distribution was 22.37%. Taste preferences peaked at 41.39% for light and were nearly equal at 41.29% for spicy.

**Table 1:** Distribution of basic demographic characteristics in four provinces and cities in Southwest China (*n* = 7,367).

Factor	Total	Yunnan	Guizhou	Sichuan	Chongqing	*p*-value
***N***	7,367	1,290	2,101	1,415	2,561	
**Gender**						0.18
Male	3,374 (48.80%)	586 (45.43%)	1,004 (47.79%)	640 (45.23%)	1,144 (44.67%)	
Female	3,993 (54.20%)	704 (54.57%)	1,097 (52.21%)	775 (54.77%)	1,417 (55.33%)	
**Age (years)**	35.48 (14.41)	36.94 (12.80)	33.87 (13.56)	35.49 (14.65)	36.07 (15.56)	<0.001
**BMI**	22.06 (2.96)	22.26 (2.90)	22.03 (3.04)	21.88 (2.86)	22.09 (2.97)	0.008
**Nationality**						<0.001
Han	6,501 (88.24%)	1,026 (79.53%)	1,714 (81.58%)	1,377 (97.31%)	2,384 (93.09%)	
Minority	866 (11.76%)	264 (20.47%)	387 (18.42%)	38 (2.69%)	177 (6.91%)	
**Residence**						<0.001
Urban	4,530 (61.49%)	794 (61.55%)	1,223 (58.21%)	859 (60.71%)	1,654 (64.58%)	
Rural	2,837 (38.51%)	496 (38.45%)	878 (41.79%)	556 (39.29%)	907 (35.42%)	
**Long-term residents**						<0.001
No	1,520 (20.63%)	275 (21.32%)	468 (22.28%)	358 (25.30%)	419 (16.36%)	
Yes	5,847 (79.37%)	1,015 (78.68%)	1,633 (77.72%)	1,057 (74.70%)	2,142 (83.64%)	
**Occupation**						<0.001
Animal husbandry and fishery	1,300 (17.65%)	193 (14.96%)	408 (19.42%)	237 (16.75%)	462 (18.04%)	
Student	1,648 (22.37%)	168 (13.02%)	408 (19.42%)	305 (21.55%)	767 (29.95%)	
Self-employed individual/Sales	1,711 (23.23%)	372 (28.84%)	591 (28.13%)	290 (20.49%)	458 (17.88%)	
Freelance	1,189 (16.14%)	191 (14.81%)	316 (15.04%)	232 (16.40%)	450 (17.57%)	
Employee	1,519 (20.62%)	366 (28.37%)	378 (17.99%)	351 (24.81%)	424 (16.56%)	
**Education**						<0.001
Low	2,275 (30.88%)	345 (26.74%)	742 (35.35%)	401 (28.34%)	787 (30.73%)	
Medium	1,470 (19.95%)	294 (22.79%)	418 (19.90%)	280 (19.79%)	478 (18.66%)	
High	3,622 (49.17%)	651 (50.47%)	941 (44.79%)	734 (51.87%)	1,296 (50.61%)	
**Flavor preferences**						<0.001
Light	3,044 (41.32%)	438 (33.95%)	803 (38.22%)	640 (45.23%)	1,163 (45.41%)	
Sour	341 (4.63%)	102 (7.91%)	107 (5.09%)	49 (3.46%)	83 (3.24%)	
Sweet	359 (4.87%)	77 (5.97%)	108 (5.14%)	50 (3.53%)	124 (4.84%)	
Spicy	3,042 (41.29%)	567 (43.95%)	937 (44.60%)	574 (40.57%)	964 (37.64%)	
Salty	581 (7.89%)	106 (8.22%)	146 (6.95%)	102 (7.21%)	227 (8.86%)	
**Hypertension**						0.008
No	6,841 (92.86%)	1,169 (90.62%)	1,959 (93.43%)	1,322 (93.24%)	2,391 (93.36%)	
Yes	526 (7.14%)	121 (9.38%)	142 (6.57%)	93 (6.76%)	170 (6.64%)	
**Hyperlipidemia**						<0.001
No	7,081 (96.12%)	1,208 (93.64%)	2,043 (97.24%)	1,372 (96.96%)	2,458 (95.98%)	
Yes	286 (3.88%)	82 (6.36%)	58 (2.76%)	43 (3.04%)	103 (4.02%)	
**Diabetes**						
No	7,182 (97.49%)	1,253 (97.13%)	2,068 (98.43%)	1,385 (97.88%)	2,476 (96.68%)	
Yes	185 (2.51%)	37 (2.87%)	33 (1.57%)	30 (2.12%)	85 (3.32%)	0.001
**Gout**						
No	7,148 (97.13%)	1,250 (96.90%)	2,035 (96.86%)	1,379 (97.46%)	2,484 (96.99%)	
Yes	219 (2.97%)	40 (3.10%)	66 (3.14%)	36 (2.54%)	77 (3.01%)	0.75

Data are presented as mean (SD) for continuous measures, and *n* (%) for categorical measure.

### Knowledge, attitudes, practices responses

In terms of KAP scores, Guizhou scored lower than Sichuan and Chongqing in the total KAP score and the knowledge section, and the difference was significant (Table 2). Guizhou received the lowest total KAP score (65.80 ± 8.17) compared with other provinces and cities, with a statistically significant difference (*p* < 0.001). In terms of knowledge component scores, Guizhou province scored lower (30.75 ± 31.68) than the other provinces and cities (*p* < 0.001). In the attitudes component score, the score of Guizhou province (69.49 ± 13.68) was lower than those of Sichuan and Yunnan, with a statistically significant difference (*p* < 0.001). Among the practices component scores, Guizhou province scored significantly lower than the other provinces and cities in the total KAP score (*p* < 0.001). In fact, it scored significantly lower than Sichuan and Chongqing (*p* < 0.001).

**Table 2:** Knowledge, attitude, and behavior scores of four provinces in Southwest China.

Factor	Total	Yunnan	Guizhou	Sichuan	Chongqing	F/x2	*p*-value
Mean ± SD							
KAP total score	67.62 (8.19)	67.15 (7. 98)^b^	65.80 (8. 17)^a^	68.75 (7. 78)^c^	68.72 (8. 23)^c^	62.09	<0.001
Knowledge	35.86 (32.77)	42.14 (32. 89)^c^	30.75 (31. 68)^a^	40.40 (34. 13)^c^	34.37 (31. 99)^b^	44.45	<0.001
Attitude	70.44 (13.88)	71.42 (13. 84)^b^	69.49 (13. 68)^a^	71.30 (13. 84)^b^	70.26 (14. 04)^ab^	7.39	<0.001
Practice	70.30 (10.39)	68.44 (9. 90)^a^	68.53 (10. 43)^a^	71.14 (9. 79)^b^	72.23 (10. 52)^c^	68.45	<0.001

Mean ± SD, Average ± Standard Deviation; In two-by-two comparisons, a difference containing different letters indicates a statistically significant difference, while a difference containing the same letter indicates no statistically significant difference.

### Single-factor analysis

The study found statistically significant differences (*p* < 0.05) in demographic characteristics such as province, gender, age, BMI, ethnicity, place of residence, occupation, education, taste preference, and prevalence of chronic diseases (hypertension, hyperlipidemia, diabetes mellitus, and gout) based on the results of the univariate analysis in Table 3.

**Table 3:** Analysis of KAP scores for different socio-demographic characteristics.

Factor	Standardized KAP total score (Mean ± SD)	F/t	*p*-value
**Province**		62.09	<0.001
Yunnan	67.15 (7. 98)^b^		
Guizhou	65.80 (8.17)^a^		
Sichuan	68.75 (7.78)^c^		
Chongqing	68.72 (8. 23)^c^		
**Gender**		−2.87	<0.001
Male	67.32 (8.46)		
Female	67.87 (7.94)		
**Age**		25.36	<0.001
18–25 years	68.57 (8.53)^b^		
26–45 years	67.06 (8.12)^a^		
>45 years	67.26 (7.75)^a^		
**BMI**		15.76	<0.001
Underweight	68.27 (8.27)^b^		
Normal	67.86 (8.11)^b^		
Overweight or obesity	66.72 (8.27)^a^		
**Nationality**		4.09	<0.001
Han	67.76 (8.16)		
Minority	66.55 (8.32)		
**Residence**		−4.93	<0.001
Village	67.02 (8.35)		
Urban	67.99 (8.06)		
**Long-term residents**		−0.60	0.547
Yes	67.59 (8.21)		
No	67.73 (8.10)		
**Occupation**		41.96	<0.001
Animal husbandry and fishery	67.38 (7.86)^b^		
Student	69.45 (8.28)^c^		
Self-employed individual/Sales	66.09 (8.19)^a^		
Freelance	66.77 (8.21)^ab^		
Employee	68.22 (7.91)^b^		
**Education**		56.45	<0.001
Low	66.54 (8.09)^a^		
Medium	66.78 (8.23)^a^		
High	68.64 (8.11)^b^		
**Flavor preferences**		5.36	<0.001
Light	67.38 (7.89)^a^		
Sour	66.57 (8.39)^a^		
Sweet	67.23 (8.97)^a^		
Spicy	67.81 (8.17)^ab^		
Salty	68.73 (9.01)^b^		
**Hypertension**		−2.73	0.006
No	67.67 (8.19)		
Yes	66.68 (8.09)		
**Hyperlipidemia**		2.01	0.044
No	67.66 (8.16)		
Yes	66.66 (8.90)		
**Diabetes**		3.05	0.002
No	67.66 (8.15)		
Yes	65.80 (9.19)		
**Gout**		2.89	0.004
No	67.67 (8.13)		
Yes	66.04 (9.84)		

Mean ± SD, Average ± Standard Deviation; In two-by-two comparisons, a difference containing different letters indicates a statistically significant difference, while a difference containing the same letter indicates no statistically significant difference.

### Multi-variable analysis

Multifactorial analysis was conducted using multiple linear regression models, and subgroup analyses were carried out after dividing the Southwest region by province. Guizhou province had significantly lower scores for KAP (*β* = −1.40; 95%CI: −1.96 to −0.84) (Table 4). Furthermore, low scores were obtained for minorities (*β* = −0.65; 95%CI: −1.23 to −0.07) (Table 4). Moreover, groups involved in self-employment and sales occupations (*β* = −1.56; 95%CI: −2.17 to −0.94) as well as freelance (*β* = −0.95; 95%CI: −1.59 to −0.31) were at a higher risk for KAP scores (Table 4). The higher education level of the group was a favorable factor for the KAP score (*β* = 1.16; 95%CI: 0.56–1.75) (Table 4). Diabetic patients were highly likely to score low on oil and salt-related KAP performance (*β* = −1.31; 95%CI: −2.51 to −0.12) (Table 4). Significant variations in KAP scores were found between urban and rural areas in Yunnan (*β* = 0.59; 95%CI: 0.19–0.98), Sichuan (*β* = 0.84; 95%CI: 0.43–1.26), and Chongqing (*β* = 0.85; 95%CI: 0.44–1.25), with residents living in urban areas having higher KAP scores (Table 5). In Yunnan (*β* = 1.33; 95%CI: 0.61–2.05), Sichuan (*β* = 1.14; 95%CI: 0.42–1.85), and Chongqing (*β* = 1.24; 95%CI: 0.52–1.96), groups that favored salty tastes had favorable KAP scores (Table 5).

**Table 4:** Relationship between socio-demographic characteristics and overall KAP scores of the four provinces in the Southwest region (*β*, 95%CI).

Factor	Southwestern China
	Standardized KAP total score
Province	
Yunnan	0
Guizhou	−1.40 (−1.96, −0.84)***
Sichuan	1.19 (0.57, 1.81)***
Chongqing	1.07 (0.52, 1.62)***
Gender	
Male	0
Female	0.29 (−0.09, 0.66)
Age	
18–25 years	0
26–45 years	−0.09 (−0.69, 0.51)
>45 years	0.44 (−0.26, 1.14)
BMI	
Underweight	0
Normal	0.04 (−0.58, 0.65)
Overweight or obesity	−0.64 (−1.35, 0.06)
Nationality	
Han	0
Minority	−0.65 (−1.23, −0.07)*
Residence	
Village	0
Urban	0.80 (0.40, 1.20)***
Occupation	
Animal husbandry and fishery	0
Student	0.69 (−0.14, 1.51)
Self-employed individual/Sales	−1.56 (−2.17, −0.94)***
Freelance	−0.95 (−1.59, −0.31)**
Employee	−0.34 (−1.05, 0.38)
Education	
Low	0
Medium	−0.07 (−0.49, 0.64)
High	1.16 (0.56, 1.75)***
Flavor preferences	
Light	0
Sour	−0.81 (−1.72, 0.09)
Sweet	−0.40 (−1.29, 0.48)
Spicy	0.26 (−0.16, 0.68)
Salty	1.29 (0.58, 2.00)***
Hypertension	
No	0
Yes	−0.31 (−1.08, 0.47)
Hypertension	
No	0
Yes	−0.58 (−1.57, 0.42)
Diabetes	
No	0
Yes	−1.31 (−2.51, −0.12)*
Gout	
No	0
Yes	−0.98 (−2.07, 0.11)
**Constant**	66.70 (65.64, 67.76)

*β*, regression coefficient; CI, confidence interval; *p < 0.05, **p < 0.01, ***p < 0.001. Bolded values denote statistically significant data at the *p* < 0.05 level.

**Table 5:** Relationship between socio-demographic characteristics and KAP scores by province in four southwestern provinces (Yunnan, Guizhou, Sichuan, and Chongqing) (*β*, 95%CI).

Factor	Standardized KAP total score			
	Yunnan	Guizhou	Sichuan	Chongqing
Gender				
Male	—	—	—	0
Female	—	—	—	0.33 (−0.05, 0.72)
Age				
18–25 years	—	0	0	0
26–45 years	—	0.22 (−0.38, 0.81)	0.09 (−0.51, 0.69)	0.17 (−0.43, 0.77)
>45 years	—	1.00 (0.32, 1.69)**	0.61 (−0.07, 1.28)	0.81 (0.12, 1.50)*
BMI				
Underweight	0	—	—	0
Normal	−0.17 (−0.78, 0.44)	—	—	0.11 (−0.51, 0.73)
Overweight or obesity	−1.04 (−1.72, −0.36)**	—	—	−0.63 (−1.34, 0.08)
Nationality				
Han	—	—	—	0
Minority	—	—	—	−1.25 (−1.82, −0.67)***
Residence				
Village	0	—	0	0
Urban	0.59 (0.19, 0.98)**	—	0.84 (0.43, 1.26)***	0.85 (0.44, 1.25)***
Long-term residents				
Yes	—	—	0	—
No	—	—	0.04 (−0.44, 0.51)	—
Occupation				
Animal husbandry and fishery	—	0	0	0
Student	—	1.41 (0.59, 2.23)**	1.24 (0.42, 2.07)**	1.11 (0.28, 1.93)**
Self-employed individual/sales	—	−1.44 (−2.04, −0.84)***	−1.67 (−2.28, −1.05)***	−1.70 (−2.32, −1.08)***
Freelance	—	−0.71 (−1.35, −0.07)*	−0.90 (−1.54, −0.25)**	0.91 (−1.55, −0.26)**
Employee	—	−0.08 (−0.79, 0.63)	−0.29 (−1.01, 0.42)	−0.33 (−1.05, 0.39)
Education				
Low	0	0	0	0
Medium	0.10 (−0.45, 0.64)	0.36 (−0.21, 0.92)	0.23 (−0.34, 0.80)	0.22 (−0.35, 0.79)
High	1.83 (1.38, 2.29)***	1.61 (1.01, 2.20)***	1.39 (0.79, 1.99)***	1.36 (0.76, 1.96)***
Flavor preferences				
Light	0	—	0	0
Sour	−1.16 (−2.07, −0.25)*	—	−1.15 (−2.06, −0.24)*	−1.06 (−1.97, −0.15)*
Sweet	−0.64 (−1.53, 0.25)	—	−0.61 (−1.50, 0.28)	−0.58 (−1.46, 0.31)
Spicy	0.07 (−0.34, 0.49)	—	0.06 (−0.36, 0.49)	0.13 (−0.29, 0.56)
Salty	1.33 (0.61, 2.05)***	—	1.14 (0.42, 1.85)**	1.24 (0.52, 1.96)**
Hypertension				
No	—	0	—	—
Yes	—	−0.59 (−1.37, 0.19)	—	—
Hypertension				
No	—	0	—	—
Yes	—	−0.72 (−1.72, 0.28)	—	—
Diabetes				
No	—		—	0
Yes	—		—	−1.28 (−2.47, −0.08)*
Gout				
No	—		—	0
Yes	—		—	−1.12 (−2.22, −0.03)*
**Constant**	66.66 (65.95, 67.36)	66.61 (65.88, 67.34)	66.44 (65.66, 67.22)	66.50 (65.55, 67.45)

*β*, regression coefficient; CI, confidence interval; *p < 0.05, **p < 0.01, ***p < 0.001; —, Variables that were not significant in the univariate analysis of each group were not included in the multivariate analysis. Bolded values denote statistically significant data at the *p* < 0.05 level.

## Discussion

Our cross-sectional survey investigated the current status of oil and salt intake knowledge, attitudes, and practices of residents in Southwestern China and their influencing factors. Overall, our study showed that Guizhou Province among the four southwestern provinces performed poorly in terms of KAP scores for oil and salt intake, which were not only subject to regional differences in terms of provinces but were also associated with ethnicity, urban/rural residence, occupation, education level, taste preference, and chronic disease prevalence. In the results of the provincial subgroup analysis, we found urban–rural differences in Yunnan, Sichuan, and Chongqing, but not in Guizhou. Among taste preferences, salty was a favorable factor for KAP scores.

Compared with the attitudes and practices dimensions, the score of the knowledge dimension of oil and salt intake is relatively low in Southwestern China. The possible reason may be that although residents pay attention to a light diet, they do not know the specific usage limits. This reflects the deficiency in the dissemination of limited-quantity knowledge. Given that most of the cooking behaviors with Chinese characteristics involve adding oil and salt by oneself during the cooking process, quantification is somewhat difficult (28). Therefore, publicity and education can be carried out in the form of teaching the usage methods of salt-restriction spoons and oil-restriction pots as well as the calculation methods of salt and oil contents on food labels. Guizhou Province exhibited inadequate performance in terms of KAP aspects about oil and salt intake. According to a survey conducted on the understanding of Chinese dietary guidelines, participants from Guizhou Province exhibited relatively lower familiarity with the dietary guidelines for individuals in China, consistent with the present findings (29). Investment in health policy planning and health education may vary from place to place due to different levels of economic development (30). Guizhou, as an economically underdeveloped area, has a relatively weak investment in primary health care (31). A pertinent study demonstrates that the standard of fundamental public health services in Guizhou Province is subpar, and substantial impediments exist in the progress of basic public health services (32). Hence, Guizhou Province exhibits a relatively low level of overall KAP, which may reflect the need for well-directed interventions aimed at enhancing dietary habits among its residents. In the future, Guizhou Province must enhance its efforts to promote the “three reduction and three health” (reduce salt, reduce oil, reduce sugar and healthy mouth, healthy weight, healthy bones) (33) and other relevant fundamental public health services compared with other states.

Education beyond high school is linked with high KAP scores. Further education allows individuals to enhance varied skills, such as augmented learning efficiency, cognitive and problem-solving skills, and better self-regulation (34). Additionally, individuals with higher levels of education frequently exhibit greater self-care awareness and a stronger inclination to obtain dietary knowledge (35). Therefore, individuals with higher levels of education are more inclined to comprehend nutritional information and emphasize the health hazards of excessive oil and salt consumption for self-regulation, consistent with past research (36, 37). Conversely, the health awareness of less educated individuals was comparatively weaker because of their constrained capacity to understand and value health-related information (38). When implementing health education initiatives, approaches should be customized according to the specific attributes of the target audience. For instance, a more vivid, intuitive, and understandable form of education should be used when targeting groups with lower education levels.

The urban–rural differences observed in Sichuan, Chongqing, and Yunnan were consistent with previous research. A previous China Health and Nutrition Survey (CHNS) reported inferior performance in diet-related KAP in rural areas compared with urban regions (39). Rural–urban disparities in dietary patterns were observed in studies conducted by Gao et al. and He et al. (40, 41). Limited access to health education resources could be a reason for these disparities. Rural areas may face greater obstacles than urban areas in obtaining health information and consulting with expert healthcare professionals due to factors, such as geographic transportation (42). Furthermore, rural inhabitants show lower proficiency in accessing health information online, particularly concerning high-speed Internet access, in contrast to their urban counterparts (43). This capacity restraint may serve as a significant obstruction to the access and utilization of health-related information by rural residents.

This study found that people with a preference for salty taste have a relatively good performance in terms of KAP regarding oil and salt intake. However, as of now, there is no research directly confirming this finding. Based on this, we conduct the following discussions and speculations. Taste has long been thought to play a crucial role in eating behavior (44). Studies have shown that individuals tend to prefer certain taste, leading to higher consumption of such food (45, 46). Numerous studies also indicate that an excessive oil and salt diet is significantly associated with an increased incidence of chronic diseases (47–49). Moreover, high-salt and high-fat diets may interact through different mechanisms and jointly promote the development of hypertension and hyperlipidemia (50, 51). Therefore, people who have long maintained a high-oil and high-salt diet are likely to be aware of the disease risks that this eating habit may bring. Maintaining dietary regulation goals helps people resist food temptations and achieve self-control intention (52, 53). Based on the above viewpoints, we can reasonably speculate that among people with a preference for salty taste, if someone is fully aware of the risks of excessive oil and salt intake and takes active measures to limit oil and salt intake, then they are more likely to obtain better KAP scores in terms of oil and salt intake. This is because the awareness of risks will prompt them to actively acquire more knowledge about healthy eating, and then pay more attention to reasonably controlling salt intake in terms of attitude, and finally achieve a reduction in salt intake in practical behavior. In addition, family factors are also key points that cannot be ignored. Family members may monitor and manage individuals with a penchant for salty foods, thereby enhancing knowledge of healthy eating and promoting healthy eating behavior. A previous study pointed out that cooking classes are crucial to the influence of housemakers in promoting healthy eating, which can help reduce the level of oil and salt intake of the whole family (54). Educating family members about the dangers of excessive salt intake through a range of means, including primary healthcare providers and home cooks, can significantly enhance knowledge and ingrained behavior (55).

The reasons for the relatively low KAP scores regarding oil and salt intake among diabetic patients are discussed below. First, diabetic patients may place greater emphasis on controlling sugar intake in their daily diet but lack sufficient awareness of the impact of salt and fat intake. Furthermore, research has found common misconceptions in the dietary habits of diabetic patients, such as the belief that plant-based oils can be consumed without restriction, which may negatively affect their dietary management and overall health (56). Additionally, some studies have pointed out that low-sodium diets might increase the risk of adverse cardiovascular events in diabetic patients (57). Moreover, a study on dietary factors in diabetic patients found that the majority of respondents were unable to adhere to diabetes dietary recommendations due to incomes below the basic wage level (58). Base on this, the following measures could be taken to improve the situation. First, providing accurate dietary information and guidance can help diabetic patients correct misconceptions about oil and salt intake, clearly informing them of the appropriate consumption levels of various types of fats and the proper way to maintain a low-sodium diet, thereby correcting their misunderstandings. Secondly, considering patients’ economic conditions and personal preferences, personalized dietary advice should be provided. For patients with lower incomes, recommending affordable food choices and cooking methods that meet the dietary requirements for diabetes can help them make healthier oil and salt intake choices while ensuring balanced nutrition within a limited budget.

In the present study, independent operators, sales personnel, and freelance practitioners had lower scores in KAP related to oil and salt intake due to the increased work pressure and uncertainty in their work hours. Research suggests that young individuals opt for fast and convenient food, such as takeout or pre-packaged meals, due to their uncertain work schedules (59). Maintaining longer working hours has been suggested to result in a lack of time for self-care and health maintenance in this population (60). This phenomenon could be a reason for their inability to pay adequate attention to healthy eating and nutritional knowledge. Irregular working hours may contribute to their preference for convenience foods and takeaways, which are generally high in fats and salts. Compared with conventional employees, those with more flexible working arrangements may experience a lack of social support from a fixed workplace. In a relatively fixed workplace environment, organizations may cover some healthcare services while supporting employees to assess their health status and take responsibility for their health by promoting a healthy lifestyle (61, 62). For self-employed individuals, salespeople, and freelance practitioners, the absence of this mechanism can lead to a sense of isolation when developing and maintaining healthy eating habits, ultimately hindering their access to healthcare and health education. In the future, this population should be reached out to optimize the allocation and utilization of health education resources.

Southwestern China is home to many ethnic minority groups, representing almost half of the country’s ethnic minority population (63). These diverse groups have unique dietary behavior and habits. Research suggests that ethnic minority populations are in a critical stage of nutritional transition and are generally less educated and health-conscious (64). Ethnic minorities face challenges when establishing or sustaining healthy eating routines during nutritional transition. This finding might explain the lower KAP scores among this group.

## Strengths and limitations

This initial cross-sectional analysis in Southwestern China employs a population survey to explore KAP regarding oil and salt intake. The strength of this study is its concentration in Southwestern China, where high oil and salt dietary habits are common. It investigates the current status of KAP of oil and salt intake among residents in this area, providing valuable support for the further development of health education initiatives aiming to reduce salt, sugar, and oil consumption. Additionally, the study provides a foundation for the advancement of quantitative measures related to oil and salt intake. However, this study has certain limitations. The survey did not incorporate the Tibet region, which is classified within the Southwest area of China’s administrative planning. The reasons are due to transportation influences and other factors related to the COVID-19 outbreak. The data situation in the Tibetan region should be bolstered to accurately portray the cognizance of oil and salt intake in the southwest region of China. The utilization of convenience sampling significantly limits the representativeness of the findings in the population. Third, despite the use of face-to-face questionnaires by the investigator, all responses were self-reported by the participants, potentially leading to recall bias. Fourth, the cross-sectional research design does not allow for the inference of causal relationships.

## Conclusion and policy recommendations

This study primarily aims to assess the current level of KAP related to oil and salt intake among residents in Southwestern China and explore influencing factors. Based on the findings, in Guizhou Province, residents exhibited the worst performance in terms of the KAP about oil and salt. The influencing factors of KAP scores related to oil and salt included the region in the province, ethnicity, rural or urban residence, level of education, and taste preferences. Due to differences in economic development levels and dietary habits among various provinces and cities, residents in different regions have different levels of awareness of oil and salt. Future health education programs should be adjusted according to the unique circumstances of each region. The government needs to promote relevant professionals to carry out dietary health education and conduct publicity in forms such as teaching the usage methods of salt-restriction spoons and oil-restriction pots and the calculation methods of salt and oil contents on food labels. At the same time, opportunities for rural residents to access modern communication technologies and experts should be increased because rural areas have relatively limited access to knowledge about oil and salt. For companies or communities where employees have irregular working hours, accessible nutritional health resources and information should be promoted to help maintain healthy dietary habits. In subsequent studies, quantitative means can be utilized to conduct a further assessment of the specific situation of oil and salt intake in Southwestern China. At the same time, intervention measures can be employed to control oil and salt intake and combine it with the incidence status of cardiovascular diseases in Southwestern China to carry out more systematic and in-depth research.

## Data Availability

The raw data supporting the conclusions of this article will be made available by the authors without undue reservation.
